# Letter from Professor Martins

**Published:** 1840-05-30

**Authors:** 


### FOREIGN CORRESPONDENCE.

### LETTER FROM PROFESSOR MARTINS.

### No. VIII.

*On Perforations of the Lungs.* By Charles Martins, D. M. P.

*Paris, April* 4, 1840.

To the Editors of the Medical Examiner.

In my last letter I gave you an account of a case of pneumo-hydrothorax with perforation of the bronchiæ, read before the Society of Medical Observation. In the debate to which the paper gave rise, all the speakers, with the exception of the author, attributed the perforation of the lung to the softening of a tubercle. This opinion I cannot adopt, but agree with the view presented by M. Legendre. What, in fact, was the amount of argument urged in opposition to him? That no instance was known of perforation of the lung, the result of empyema. This was the sum of what was advanced on the opposing side of the question. The discussion on this subject brought to my recollection a thesis entitled, "An Essay on Perforations of the Lungs, submitted by M. Vigier Devarennes to the Faculty of Medicine, in 1834, numbered 43." The author brings successively under notice the various diseases which may occasion perforation. They are: 1st, tubercles, passed into the state of suppuration; 2dly, severe hæmorrhages, causing a laceration of the pulmonary tissue. There are three such cases well authenticated and analysed by M. Cruveilhier, in the article Apoplexy, in the Dictionary of Practical Medicine and Surgery. 3dly, pulmonary empyema. Littre, Meckel, Cruveilhier, Devillier, and W. Hewson report instances in which the rupture of one or more vesicles established a communication between the pleura and bronchiæ. 4thly, acephalocystous cysts, (Clinique des Hopitaux, No. 5;) 5thly, chronic pleurisy. Here, the author cites all the cases to be found on record, in which purulent collections in the pleuræ thus opened a passage across the lung. M. Cayol (Biblioteque Médicale, tom. 40,) has shown that there exists a variety of encysted empyema, often confounded with vomicæ, and which most frequently terminates by perforation. In these cases, says he, the passage is simple, rounded, straight, lined with a membrane, and without sinuosities. In the case of M. Legendre the fistula presented all the characters just described by M. Cayol. M. Legendre, in the debate, dwelt upon the fact that there were no sinuses (poches) in the neighbourhood or on the route of the fistula; whence he concluded that it could not be the product of the softening of a tubercle. M. Louis very justly observed, that similar fistulæ are sometimes destitute of sinuses; he might have added that in many cases, sinuses are found either laterally or upon the course of the passage, without the existence of tubercles. The following case will illustrate this fact; I noted it in 1833, in the service of M. Biett, and read it the same year before the Society of Medical Observation. I have thought that it might interest your readers, because it is the type of a sixth order of disease which occasions perforation of the lungs—abscesses formed in the abdominal cavity.

*Case of Abscess of the Peritoneum opening into the Bronchiæ—Gangrene of the Lung.*

The subject of this case, named Colin, aged, 28 years, a ship painter, was feeble in infancy, and walked very late. About the age of 14, he was confined to bed with a fever for fifteen days. From that time his health was tolerably good. He had never coughed nor spit blood, nor sweat in the night, nor suffered from pains between the shoulders. From the beginning of the present year, however, he experienced some slight attacks of indisposition, occasioned chiefly by colics. From the beginning of the month of May, these colics had become more frequent, and were accompanied by constipation. The patient at this time was working very unusually hard, continuing at work sometimes during the nights, and not resting even on Sundays. The 4th of May the attacks of colic became unusually severe; he swallowed a glass of brandy, and then returned home; towards four o'clock in the afternoon he ate some vegetable soup. The following day, the attacks of colic continued. They were not accompanied with looseness of the bowels, but with a sensation of heat and pain, increased by pressure. The belly was not tumefied; the patient went to stool only by the aid of injections and passed his fœces in very small pieces.

On the eighth, he sent for Dr. Jolly, who recognised a well-marked case of lead colic. He ordered fifteen leeches to be applied to the abdomen, with hip baths, with ptisan of marshmallows, and injections. This treatment was followed by relief, but, on the sixteenth, Colin was suddenly taken with a severe pain in the right flank at the level of the umbilicus, increased by pressure and respiration. The pain shot through the entire right side as far up as the shoulder-blade; a jaundiced tint spread over the whole body. The pain went on constantly increasing, as well as the oppression; sixteen leeches were applied to the side. The patient was relieved, but the pain advanced towards the epigastrium. On the 23d, the cough increased, in the midst of a violent fit of which, occurred the sudden expectoration of a large quantity of white pus, accompanied by so great a fœtidity that it was necessary to sprinkle the chamber with chloride of lime. Dr. Jolly diagnosticated an abscess of the liver which had opened into the lung. He had a large blister applied to the side.

The patient was brought to the hospital on the 29th of May. He was then in the following condition:—Face pale and thin; eyes projecting; a slightly jaundiced tint in the conjunctiva; tongue moist, slightly brown in the middle; breath fœtid. Clavicles prominent; ribs projecting under the skin. The right inferior portion of the thorax strikingly swollen in front. Anteriorly, percussion sonorous in the entire *left* side, above and below the clavicle; stomachal sound from the sixth rib downwards towards the epigastrium. Respiration puerile, with well-marked vesicular expansion. Sounds and impulse of the heart normal. In the entire lateral region of the chest, perfect sonorousness, with slight mucous rhonchus: on the *right*, extreme sonorousness; respiratory murmur very feeble to the extent of three inches, ascending from the nipple. Elsewhere, ordinary sonorousness, respiration feeble, marked with mucous rhonchus. In the lateral portion, flatness, increasing from the armpit to the region of the liver, where it is complete; at the level of the nipple mucous rhonchus is heard, and a grating sound like that produced by the friction of leather. An externe of the ward asserts that he heard the metallic tinkling, and compares the sound to that which would be produced by the collision of two five-franc pieces. No resonance of the voice. Posteriorly, flatness of the two sides, but especially of the right, at the internal portion of the shoulder blade. Here, in a space of three inches in height, the sound is entirely flat. Well-marked tubal respiration is heard, accompanied with bronchophony. Throughout the rest of the lung on this side, very strong mucous rhonchus. On the *left*, mucous rhonchus masking the respiratory sound throughout the entire lung. The respiration is difficult, and accelerated by the least movement. When the patient coughs, he expectorates at the same time a quantity of liquid equivalent to about a spoonful of soup. This expectorated liquid is homogeneous, of a brownish-red colour. It resembles a mixture of pus and blood; the odour which it exhales is extremely fœtid, and resembles that proceeding from animal matter in a state of putrefaction. Sometimes it is insipid and nauseous like that of wet plaster. Abdomen slightly tender, tympanitic, giving a clear sound on percussion. It is not painful at any point, even on pressure.

*28th, morning.*—Oppression less severe; respiration less accelerated. Pulse ninety-five; face flushed; stools natural. The day before he had expectorated gray sputa, frothy, with the odour of wet plaster still sensible, but less characterized than at the period of the patient's entrance; forty-nine inspirations per minute; tongue moist, rosy in the middle, a little foul at the edges. The grating leather-sound continues posteriorly and below the right nipple. *Posteriorly on the right*, flatness throughout the whole extent of the lung; sub-crepitant and mucous rhonchus, accompanied with bronchial respiration. On the *left*, mucous rhonchus, with some sibilant; a little bronchophony above. Death at four o'clock in the evening.

*Autopsy, the 30th of May.*—Body emaciated, pale, spotted with blue, posteriorly; articulations stiff; abdomen slightly tympanitic; muscles red and tolerable fleshy. Upon opening the abdomen, we perceived a vast cavity which extended between the ribs and liver; the latter was considerably pushed in, and separated from the abdominal parietes by a pint of grayish and grumous pus. The cavity of the abscess extended from the fourth to the ninth rib, in about a length of half a foot; its form was that of a triangle, or of a cone, with the base above. From the liver to the ribs, there was a distance of about four inches, which diminished towards the lower part, where the liver was in contact with the muscles of the abdomen. These parietes of the abscess were thus formed: superiorly by the diaphragm, externally by the ribs and the soft part covering them; inferiorly by the intestinal circumvolutions, and within by the convex surface of the great lobe of the liver. This cavity was throughout lined with a false membrane of the thickness of a fifth to an eighth of a line, yellow and tolerably adherent to the subjacent parts. The peritoneum was every where intact, except in a single point. At the most convex portion of the diaphragm, for a space of three inches in length by two in breadth, this membrane presented an unequal appearance of mixed white and brown, covered with very long filaments. The edge of this space is red, projecting, unequal, and presents the appearance of a vast intestinal ulceration. This surface is formed at the expense of the diaphragm, the peritoneum, and the base of the lung; the latter is bare for the extent of an inch and a half in length by the same in breadth. This was ascertained in the following manner: A syringe full of water was fitted to the trachea; the piston was hardly touched before the liquid appeared on this surface, chiefly by two principal points. Thus, the pulmonary tissue, and even the extremity of the bronchial tree, were shown to communicate with the purulent centre. Around this point, the diaphragm had lost its muscular appearance, and was indurated.

The trachea was internally of a greenish white, but from its bifurcation to the extreme bronchial ramifications, were found well marked red spots.

*Right Lung.*—Superior lobe red, crepitating, giving out, upon being cut, a frothy and bloody liquid. At the posterior superior portion of the inferior lobe, is observed a large yellowish spot, dotted with red, three inches in height by two in breadth. It is perceived externally that this spot corresponds to a cavity, and forms a very thin wall. Upon incising it, a sinus is cut into, very winding, divided into several secondary cavities, which, united, are about equivalent in volume to a goose's egg. It contains about three ounces of a grayish, homogeneous liquid, of a very fœtid, gangrenous odour. Numerous bands traverse this cavity and constitute anfractuosities; these bands are formed by the bronchiæ and vessels, the external surface of which is protected by a false membrane, the fifth of a line in thickness; at the interior of the vessels are found coagula of blood. The gangrenous cavity is separated from the diaphragmatic ulceration, by a space of about three inches, so that there is between them no direct communication. The bronchiæ which open into the sub-diaphragmatic purulent collections do not cross the cavity, but, after a circuit of half an inch, join the large bronchial tubes which communicate with it. Thus, liquids proceeding from the gangrenous cavity and the purulent collection, can easily mix. The remainder of the inferior lobe is of a brownish-red colour, and sinks in water. The anterior portion of the right lung is red, crepitating, and hard. In the region corresponding to the mammæ, the vesicles are very much developed.

*Left Lung.*—Superior portion bluish externally, of a rosy red internally, crepitating, giving out a liquid slightly tinged with blood. At the summit is a little cavity, about the size of a walnut, and entirely similar to the gangrenous cavity, observed in the right lung. The inferior posterior portion is of a red colour, does not crepitate, and scarcely floats on the surface of water. This whole portion is adherent to the costal portion, and covered with a slight false membrane. The internal surface of the pulmonary veins contains neither blood nor pus.

*Liver.*—The whole superior and external portion of the great lobe, to within an inch of the free edge, instead of being convex, has its surface flat, forced backwards towards the vertebral column, and pitted with superficial depressions. It is lined by a yellowish false membrane of the kind already described. Upon cutting into the liver, its tissue, for a thickness of four lines, is found of a brownish slate colour, while the remainder is of a nearly uniform pale red, and of a normal consistence. This brown colour is not detected in the lesser lobe, which is not covered by a false membrane. At the inferior and external point of the great lobe, and upon the inferior surface, is a small oval false membrane, an inch in diameter; it is a quarter of a line thick and entirely isolated; below it, the liver presents a grayish slate coloured tint. This false membrane served as a wall to an abscess of the size of an egg, elsewhere circumscribed by the convolutions of the intestines covered with a false membrane. Two purulent collections extended through the abdomen, one between the cœcum and the abdominal parietes, down to the anterior superior process of the os ileum; the other between the colon and the mass of small intestines to the groin. The gall-bladder contains a yellowish bile, with a bistre-coloured matter, becoming soft between the fingers. No traces of abscess in the parenchyma of the liver. Spleen healthy. Slight adhesion between the intestinal circumvolutions through the medium of little false membranes. Stomach and intestines healthy. The coagula taken from the large vessels were firm, black, and without appearance of pus. Brain healthy. It is evident that the perforations preceded the formation of the gangrenous abscesses. This is inferred from the whole history of the disease, and, particularly, from the symptoms observed the 29th of May.

In the same essay is found a case altogether analogous, observed by M. Piet, and reported at length by M. Vigier, after mine.

To close the circle of diseases which may give rise to perforation, and which are the more usual cause of it, I add the history of a case observed by me in 1828, in the service of M. Husson, at the Hôtel Dieu.

*Phthisis—Perforation of the Lung—Metallic Tinkling.*

The subject of this case, named Bechadergué, is an old soldier, forty-eight years of age, tall, thin, and so broken down by hard usage, that he appears sixty years of age. Five years since, he had an attack of acute pneumonia, and has coughed ever since.

*Present condition*, (20*th January*, 1839.)—Face emaciated ; features altered ; cheeks red ; pulse small, frequent, and feeble. Cough difficult, feeble, accompanied by severe pain in the right side, and followed by the expectoration of greenish, viscid sputa, which adhere to the cup, and are surrounded by a small quantity of clear frothy mucus. Throughout the left anterior portion of the chest, percussion gives a clear sound, and the respiration is puerile. Comparing the two sides, the right, at the superior portion, gives a sound, which, without being flat, is much less sonorous than that of the left side. But at the level of the left nipple and below it, we meet with a hollow resonance indicating a large cavity. Upon applying the ear below the right shoulder-blade, a silvery sound is heard, similar to that which would be produced by drops of water falling one by one into an empty glass goblet. All who listened made use of the same comparison. The drops of water do not seem to fall constantly, but only at intervals, sometimes two follow each other rapidly.

24*th of January.*—The patient is very much oppressed ; the beatings of the heart are very violent, and heard throughout the whole chest. The metallic tinkling continues, and is accompanied by well-marked bronchophony.

26*th.*—The metallic tinkling is heard only when the patient coughs; it resembles the sound given out by a goblet when struck. Death the 27th.

*Autopsy.*—The chest having been opened on the right side, a very observable quantity of air escaped. A vast cavity, extending from the fourth to the last *true* rib, was half filled with a frothy and purulent liquid. In order to find the fistula, a bellows was fitted to the trachea. Every time it was blown, the lung was slightly raised, but no bubbles of air were seen to rise above the liquid in which it was immersed. A light having been introduced into the thorax, the flame remained motionless. Then the lung was detached, and at the anterior and inferior portion of the superior lobe, an opening was found, rounded, oval, with hardened edges, communicating with the bronchial ramifications by a series of little cavities. The summit of the lung, as well as the greater portion of its parenchyma, was filled with little cavities, and studded with crude tubercles. Here and there were remarked points of red hepatization. There were likewise tubercles at the summit of the left lung. The other viscera were healthy.

